# 

**Published:** 1821-07

**Authors:** 


### THE LONDON MEDICAL AND PHYSICAL JOURNAL.

CONTAINING

ORIGINAL CORRESPONDENCE OF EMINENT PRACTITIONERS,

AND

CRITICAL ANALYSIS OF NEW WORKS

RELATING TO MEDICINE, SURGERY, MIDWIFERY, CHEMISTRY, PHARMACY, BOTANY, AND NATURAL HISTORY.

---

Successively conducted by

[illegible]ADLEY, M.D.

[illegible] M. WILLICH, M.D.

[illegible]TTY, M.D.

[illegible] A. NOEHDEN, M.D.

DRS. ARNEMAN AND PFAFF,

J. ADAMS, M.D.

W. SHEARMAN, M.D.

W. ROYSTON, ESQ.

S. FOTHERGILL, M.D.

W. HUTCHINSON, M.D.

AND NOW EDITED BY

A. B. GRANVILLE, M.D. F.R.S. F.L.S. M.R.I.

Physician in Ordinary to his Royal Highness the Duke of Clarence; Licentiate of the Royal College of Physicians; Principal Physician to the Royal Infirmary for Children; Physician-Accoucheur to the Westminster General Dispensary; Honorary Member of the Royal Academy of Medicine of [illegible]d; Foreign Associate of the Royal Academy of Sciences of Naples; Member of the Medico-[illegible]hirurgical Society of London; of the Philosophical and Literary Society of Manchester; of the Philomatic and Philotechnic Societies of Paris; Member of the Society of the Faculty of Medicine of Paris, of the Societé Medicale d'Emulation, and the Cercle Medical of the same town; Correspondent of the Prussian Physical Society of the Lower Rhine; Member of the Imperial Academy of Lucca; of the Societies of Florence, Marseilles, Leghorn, Pistoja, Padua, Venice, &c. &c.

---

VOL. XLVI.

FROM JULY TO DECEMBER, 1821.

Et quoniam variant morbi, variabimus artes;

Mille mali species, mille salutis erunt.

---

*LONDON:*

PRINTED FOR THE PROPRIETORS,

*By J. and C. Adlard, 23, Bartholomew Close;*

PUBLISHED BY J. SOUTER, 73, ST. PAUL'S CHURCH-YARD;

AND MAY BE HAD OF ALL BOOKSELLERS.

W1
LO 31

### ANALYTICAL TABLE OF THE CONTENTS

OF

THE FORTY-SIXTH VOLUME

OF THE

### London Medical and Physical Journal.

### DESCRIPTIVE ANATOMY.

PAGE

Of some points in the development of the heart and intestines . . 3

Of the structure of the human lung in different ages . . . ib.

Of the lymphatic vessels of birds . . . . . . . 4

reptiles and fishes . . . . 5

Of the ultimate arrangement in the structure of the human body . . 6

Of a species of mucous follicle under the skin . . . . 8

Comparative anatomy of the brain . . . . . 81

Elementary work on Anatomy . . . . . . 416

Kidney, internal anatomy of . . . . . . 479

Testicle, descent of . . . . . . . . 481

Seminal vesicles, prostate glands, Cowper's gland . . . 482

Penis, urethra . . . . . . . . 484

### PHYSIOLOGY.

VITAL PHENOMENA, *in general.*

On the functions of the spleen . . . . . 11

Reflections on the relations of heat to vitality . . . 265

NERVOUS AND SENSITIVE SYSTEMS, *in general.*

NUTRITIVE FUNCTIONS (*organic*, of BICHAT).

*Appetite for, and administration of, food.* Fasting for three days in an infant . . . . . . . . 605

*Hunger.* Experiment on the nature of . . . . . 139

*Mastication.* Polyphagia . . . . . . . 518

*Insalivation* . . . . . . . .

*Deglutition* . . . . . . . . .

*Digestion.* Influence of galvanism on . . . . . 26

Example of rumination in man . . . . . 119

*Absorption.* Experiments to ascertain the existence of a communication between the absorbents of the intestines and the vena portarum 13

Direct communication of the spleen with the lacteals of the intestines 14

On the means of . . . . . . . 18

*Circulation* . . . . . . . . 100

Office of the thoracic duct with regard to . . . . 11

On the non-irritability of arteries . . . . . 20

Introduction of foreign substances into the . . . 21

On the injection of mercury into the . . . . 24

*Respiration* . . . . . . . . .

*Assimilation.* Re-union of divided parts . . . . 25

*Secretion.* Properties and qualities of human urine . . . 477

Semen . . . . . . . . . . 480

*Excretion* . . . . . . . . .

CALORIFIC FUNCTION . . . . . . . 266

PAGE

ANIMAL FUNCTIONS, *in general.*

Influence of water on animal substances . . . . 459, 548
Galvanic experiments on a criminal . . . . 612
*Sight.* Considerations on some points in the theory of vision . 26, 27
*Hearing.* On the physiology of . . . . . 503
*Smell* . . . . . . . . .
*Taste* . . . . . . . . .
*Touch* . . . . . . . . .

PERCEPTIONS (*Intellectual*), AND THEIR RESULTS.

*Movements* . . . . . . . .
*Sleep* . . . . . . . . .
*Voice* . . . . . . . . .

GENERATIVE FUNCTIONS.

*Proper effects of.* On hybrid productions . . . . 29
Hybrid of the mare and quagga, produced under extraordinary circumstances . . . . . . . ib.
*Preparatory to the generative act* . . . . . 480
*Gestation* . . . . . . . .
*Superfœtation.* An instance of . . . . . 31
*Parturition* . . . . . . . .
*Lactation* . . . . . . . .
*Sexual characteristics* . . . . . . .

PECULIARITIES IN THE FUNCTIONS AT THE DIFFERENT PERIODS OF LIFE.

*Fœtal life.* Nutrition of fetus . . . . . . 31
*Infancy* . . . . . . . . .
*Childhood* . . . . . . . .
*Puberty* . . . . . . . . .
*Manhood* . . . . . . . .
*Old age* . . . . . . . .

PECULIARITIES IN THE FUNCTIONS OF INDIVIDUALS.

### PATHOLOGY.

GENERAL REMARKS.

Commentaries on the Doctrine of Broussais . . . 32

OBSERVATIONS RELATIVE TO.

ABSCESS of the Female Breast . . . . . . 410

ANGINA PECTORIS . . . . . . 453, 573

ARACHNOID MEMBRANE, diseases of . . . . . 33

ARTERITIS . . . . . . . . . 529

BILIOUS COMPLAINTS . . . . . 128, 211

BLADDER, diseases of . . . . . . 491

BRAIN, diseases of . . . . . . . 34

in children . . . . . 37

protrusion of the . . . . . . 543

BRONCHOCELE . . . . . . . . 49

CALCULOUS DISEASES . . . . . . . 484

CARDITIS . . . . . . . . . 571

DELIRIUM, produced by Belladonna . . . . 120

ENLARGEMENT of the heart . . . . . . 577

EPILEPSY . . . . . . . . . 40

ERYSIPELAS . . . . . . . . 279

FRACTURES, of the cranium . . . . . 542

FUNGUS EXOSTOSIS of the scapula . . . . . 182

GANGRENE, HOSPITAL . . . . . . . 41

GASTRO-ENTERITIS . . . . . . . . 364

GOUT, pathology of . . . . . . . 134

HERNIA, PUDENDAL . . . . . . . 51

HIP-JOINT, scrofulous disease of the . . . . 413

HYDATIDS of the kidney . . . . . . 488

HYDROCEPHALUS . . . . . . . . 353

PAGE
INFLAMMATION, of intestines, in a fetus . . . . . 43
phenomena of, in general . . 226, 317
Varieties of . . . . . 327
INDIGESTION, diseases of . . . . . . 47
INTESTINES, case of obstruction . . . . . 177
LYMPHATICS, scrofulous affections of . . . . 408
MASTODYNIA, or Milk Abscess . . . . . 179
OPHTHALMIA . . . . . . . 41
PERIPNEUMONIA . . . . . . . 529
PERITONEUM, scrofulous inflammation in children . . . 497
POLYPI of the heart . . . . . . . 579
PROSTATE GLAND, scrofulous affection of . . . . 412
SCROFULA . . . . . . . . 399
SPINAL DISEASES . . . . . . 89, 415
SWELLED LEG . . . . . . . 279
TESTICLE, disease of the . . . . . . 411
TESTICLES, diseases of . . . . . . 494
TIC DOULOUREUX . . . . . 270, 284, 457
TUMORS . . . . . . . . 541
URINARY ORGANS, diseases of . . . . . 47, 476

### PATHOLOGICAL ANATOMY.

Acephalic fetuses . . . . . . . . . 52

Transposition of the thoracic and abdominal viscera . . . . 54

Osseous tumor in the spleen . . . . . . . ib.

Appearance of tuberculous lungs . . . . . . . 55

Singular ossification of the uterus . . . . . . . 56

Strangulated intestine . . . . . . . . 178

Osseous tumor of the scapula . . . . . . . . 183

Lusus naturæ (a Chinese) . . . . . . . . 259

Gall-bladder, thickened . . . . . . . . 369

Biliary calculous of large dimensions . . . . . . 370

Ossification of the ventricular aorta . . . . . . 373

Malformation of the heart . . . . . . . . 499

Hydatid in the heart . . . . . . . . 502

Transposition of the abdominal viscera . . . . . . 610

Arteritis, appearances *post mortem* in . . . . . . 533

Double fetus . . . . . . . . . 517

Singular fetus . . . . . . . . . ib.

Extraordinary case of distension of the œsophagus . . . 540

### NOSOLOGY.

Inquiry into the various Medical Doctrines of the Day, &c. . . 422

### SEMIOLOGY.

RELATIVE TO GROUPS OF SYMPTOMS, OR DISEASES.
Of Indigestion . . . . . . . 139
Scrofula . . . . . . . . . 401
Carditis . . . . . . . . . 571
RELATIVE TO PARTICULAR ORGANS, STRUCTURES, AND FUNCTIONS.
Chicken-pox, parallel between it and small-pox . . . 513

### HYGIENE.

Relative to temperature in consumption . . . . . . 115
the origin and frequency of urinary calculi . . 117, 371
the plague of Egypt . . . . 123, 207, 209
poisons . . . . . . . . 158

PAGE

Relative to vaccination . . . . . . . 255, 508
origin of syphilis . . . . . . . 257
the yellow fever . 463, 508, 520, 521, 523, 601, 604
cholera morbus . . . . . . 603, 604

### THERAPEUTICS,

GENERAL CONSIDERATIONS ON.

OBSERVATIONS RELATIVE TO

ANASARCA, use of ol. terebinthinæ in . . . . 201

CARDITIS . . . . . . . . 572

CHOREA, cured by arsenic . . . . . 499

use of ol. terebinthinæ in . . . . 199

CONVULSIONS, use of ol. terebinthinæ in . . . 199

EPILEPSIA, use of ol. terebinthinæ in . . . . 137

ERYSIPELAS, mercurial ointment in . . . . 279

FORMATION of an artificial anus . . . . . 72

FRACTURE of the pubis . . . . . . 501

GANGRENE . . . . . . . 75

GONORRHŒA, the use of cubebs in . . . . 155

GOUT, advantage of small doses of colchicum in . . 441

HEART, diseases of the . . . . . . 569

HEMORRHAGIA, use of ol. terebinthinæ in . . . 194

HERNIA, *cerebri* . . . . . . . 543

HYDROPHOBIA . . . . . . . 257, 547

HYDROCEPHALUS, compression in . . . . 343

use of ol. terebinthinæ in . . . 202

HYPOCHONDRIASIS . . . . . . . 560

HYSTERIA, use of ol. terebinthinæ in . . . . 200

INDIGESTION . . . . . . 128, 211

INFANTILE DISEASES, mismanagement of . . . 567

INFLAMMATION, treatment of, in general . . . 226

INTESTINES, obstruction of the . . . . . 273

INTOXICATION, use of ammonia in . . . . 262

LUMBAGO, use of ol. terebinthinæ in . . . . 193

MASTODYNIA, treatment of . . . . . 179

MENORRHAGIA, use of ol. terebinthinæ in . . . 196

NECROSIS . . . . . . . . 356

PARALYSIS, partial . . . . . . 278, 608

POISONS . . . . . . 161, 434, 517

RHEUMATISM, use of ol. terebinthinæ in . . . 192

SARCOCELE . . . . . . . . 71

SCROFULA, treatment of . . . . . . 399

SWELLED LEG, mercurial ointment in . . . . 279

TÆNIA, prussic acid against . . . . . 258

TETANUS . . . . . . . . 538

TIC DOULOUREUX, use of carbonate of iron in . 270, 284, 457

ULCERS, scrofulous . . . . . . . 410

URETHRA, strictures of the . . . . . . 148

use of caustic bougies in strictures of . . 492

WORMS, use of ol. terebinthinæ in . . . . 205

### CHIRURGERY.

*Respecting the Operations for*

AMPUTATION of the foot . . . . . . . 504

ANEURISM . . . . . . . . 502

BRONCHOCELE, seton in . . . . . . . 500

CUPPING . . . . . . . . . 580

HERNIA . . . . . . . . . 610

PUBIS, OS, fractured . . . . . . . 501

STONE IN THE BLADDER . . . . 373, 439, 505

URETHRA, dilatation of, by instruments . . . 148

### OBSTETRICISM.

| | PAGE |
|---|---|
| On the formation of a good nipple | 181 |
| On the female economy | 307 |
| On rupture of the uterus | 451 |
| Separation of a portion of the uterus | 506 |
| Cæsarean operation | 523 |

### MEDICAL JURISPRUDENCE AND MEDICAL POLICE.

| | |
|---|---|
| Poisoning by common sausage-meat | 66 |
| Poisoning by oxymuriate of mercury | 68 |
| On the doctrine of contagion in plague | 123, 207 |
| Lectures on Juridical Medicine | 158, 244, 337 |
| irritative poisons | 161 |
| narcotic ditto | ib. |
| narcotico-acrid ditto | ib. |
| septic ditto | ib. |
| On medical education and practice | 171, 535, 611 |
| Bills of Mortality for London, 1820 | 175 |
| Report of the Committee of the House of Commons on Plague | 209 |
| Case of poisoning by arsenic, successfully treated | 434, 466, 545 |
| Correspondence (official,) respecting the yellow-fever at Marseilles | 465 |
| Poisoning by oxymuriate of lime | 517 |
| Juridical Medicine | 522, 606 |
| Infanticide | 606 |
| Adulteration of milk | 609 |

### CHEMISTRY.

| | |
|---|---|
| A Dictionary of Chemistry, by Dr. Ure | 241 |
| Action of cork on chalybeate water | 259 |
| Decomposition of blood | 351 |
| Water, influence of, on the properties of animal substances | 459, 548 |
| Benzoic acid | 516 |
| Nitrate of potash | 517 |
| Effects of copper on vegetation | 609 |
| Effects of metallic salts on blue vegetable colours | ib. |
| Analysis of mineral waters | 610 |

### MATERIA MEDICA, PHARMACY, MEDICAL BOTANY, AND SOME POINTS IN NATURAL HISTORY.

| | |
|---|---|
| Black Pepper in Fever | 78 |
| Effects of oleum terebinthinæ on the animal economy | 107 |
| Efficacy of cubebs in gonorrhœa | 155 |
| Experiments on veratrine | 169 |
| Nerves in the mustachios of the phoca | 170 |
| Vertiginous indigestion in the horse | 172 |
| Cross breed between the common cat and the pine-martin | 173 |
| Mummy-pits in Egypt | 174 |
| On the employment of terebinthinous remedies in diseases | 185 |
| Modes of exhibition of ditto | 189 |
| Arsenic, presence of, in the mediastinum of animals poisoned by it | 259 |
| A new plant from Sumatra | 347 |
| On phosphorescence in general | 348 |
| Apple-bread | 350 |
| New property of the hydrocyanic acid | 359 |
| Note to ditto, by the Editor | 361 |

| | PAGE |
|---|---|
| Egyptian mummy, opening of an | 377 |
| On the use of colchicum | 441, 551 |
| On tar fumigation | 454 |
| Mexican Flora | 435 |
| Unicorn | 436 |
| Cathartine | 437 |
| Piperin | ib. |
| Iodine, internal use of | 438 |
| Origin of vegetables | 516 |
| Medeography | 517 |
| Tincture of the common poppy, as a substitute for laudanum | 544 |
| Zoology—Salamander | 597 |
| Milk | ib. |
| Anti-epileptic powder | 605 |
| A new gum-resin | ib. |
| Lampyrus noctiluca | 607 |
| Leech of Ceylon | ib. |
| Plant soluble in water | 609 |

### MISCELLANEA.

| | |
|---|---|
| Anniversary meeting of the Benevolent Medical Society | 170 |
| Captain Parry's Journal, extract of | 261 |
| Phosphorescence of wounds | 263 |
| Isothermal Lines, or the Laws of Distribution of Heat over the Globe | 287, 380, 468, 554 |
| Phrenological Society of Edinburgh | 343 |
| Report of the Ophthalmic Committee | 345 |
| Medical Logic | 385 |
| Report of the Central Committee of Vaccination in France | 508 |
| Medical funeral | 516 |
| Periodical medical publication in Spain | 518 |
| University of Edinburgh (Lectures) | 522 |
| Spanish apothecaries | 524 |
| Ecole Physio-Pathologique | ib. |
| Medico-Chirurgical Society of Edinburgh | 434 |
| Eye-Infirmary at Winchester | 434, 599 |
| Prize Questions | 437, 516 |
| Pharmacopœia of the United States of America | 589 |
| Royal College of Physicians in London | 597 |
| Air-pump bath | ib. |
| Liverpool Eye-Infirmary | 598 |
| New barometer | 604 |
| Dr. Bancroft | 606 |
| Mr. Wilson | 607 |
| Dr. Rigby | 612 |
| Propagation of sound in elastic fluids | 608 |
| Journal of Experimental Physiology | 611 |
| Medical promotion | 612 |
| Expression of gratitude to a physician | ib. |
| Meteorological Journals | 176, 264, 352, 439, 526, 615 |
| Monthly Catalogues of Books | 175, 263, 351, 439, 527, 615 |
| Notices to Correspondents | 440, 527, 616 |

### LIST OF PLATES.

| | |
|---|---|
| Dr. E. HARRISON's Illustration of Spinal Diseases | *to face page* 89 |
| Mr. J. HARRISON's Case of Obstruction of the Intestinal Canal | 177 |

### NAMES OF AUTHORS,

*Of whose Works, Observations, &c. either a detailed Account, or more or less general View, is given in*

### THE FORTY-SIXTH VOLUME OF THE LONDON MEDICAL AND PHYSICAL JOURNAL.

PAGE

ADAMS, Report of the Ophthalmia Committee respecting Sir WILLIAM . 344
ALARD, Dr. of *Paris*, on the Continuity of the Arterial Ramifications with those of the Veins . . . . . . . 6
ANDRAL, Mr. of *Paris*, on the Nerves which go to the Mustachios of the Phoca . . . . . . . . 170
Experiments on Veratrine, Colchicum Autumnale, and Sabadilla . 169
ARNOLD, Dr. account of a new Plant of Sumatra . . . 347
ARNOTT, Mr. JAMES, of *London*, a Treatise on Stricture of the Urethra. Cases illustrative of the Treatment of Obstructions in the Urethra 148
BALLY, Dr. of *Paris*, particulars of the Fever in Spain . . . 602
BAMPFIELD, Mr. of *London*, on a Disease of the Spleen . . . 54
On the use of small Doses of Colchicum in Gout, and Objections to the Gastro-hepatic Pathology . . . . . . 441
BANCROFT, Dr. account of his death . . . . . 606
BARTH, Mr. of *Strasburgh*, on a new Barometer . . . . 604
BARTON, Mr. JAMES, of *Birmingham*, description of a double fetus . 517
BECLARD, Dr. of *Paris*, account of his performing the Cæsarean Operation 523
BEGIN, Mr. of *Paris*, on the Doctrine of Broussais . . . 32
BELZONI, Signor, description of the Mummy-pits at Thebes . . 174
BLANE, Sir GILBERT, on the effect of Mechanical Pressure in Hydrocephalus . . . . . . . . . 353
Elements of Medical Logic . . . . . . . 385
BOND, Mr. ED. T. of *Nun Eaton*, case of Fungus Exostosis of the Scapula 182
BOULEY, Mr. of *Paris*, on some Phenomena produced by the Introduction of Air into the Veins of a Horse . . . . . . 50
BRETON, Mr. P. on the Bark of Swietenia Febrifuga, as a Substitute for that of Cinchona . . . . . . . . 503
On the Efficacy of the Bark of Pomegranate Tree, in cases of Tænia ib.
BROUSSAIS, Mr. of *Paris*, Examen des Doctrines Medicales . . 422
On his Ecole Physio-Pathologique . . . . . . 524
BROWNRIGG, Mr. Deputy Inspector, on the Air-pump Bath . . 597
BURNE, Mr. JOHN, of *Edinburgh*, Observations on Mastodynia, or Milk Abscess . . . . . . . . . . 178
BURMESTER, Mr. A. case of Tetanus, successfully treated . . 506
CAGNOLA, Dr. of *Milan*, proposals for killing the Tænia Cucurbitina by means of Prussic Acid . . . . . . . 258
CAMPBELL, Dr. of *Edinburgh*, case of Transposition of the Abdominal Viscera . . . . . . . . . . 610
CAMPBELL, Mr. the Missionary, an account of the Unicorn . . 436
CHAUSSIER, Professor, of *Paris*, on acute Peritonitis and Enteritis in a Fetus 45
CHEVREUL, Mr. of *Paris*, on the Influence of Water in Animal Substances 459,548
CHEVALIER, Mr. of *Liverpool*, a Reply to Dr. James Johnson's Letter on the Epidemia in Spain . . . . . . . . 523
CHURCHILL, Mr. of *London*, on Acupuncture . . . . 74
CLOQUET, Dr. JULIUS, of *Paris*, on Pudendal Hernia . . .
COATES, Mr. H. of *Salisbury*, case of Fractured Os Pubis . . 501
Case of Aneurism of the Carotid Artery . . . . 502
COOPER, Sir A. Extraction of numerous Calculi from the Urinary Bladder, without the employment of cutting Instruments . . 505
COPLAND, Dr. of *Walworth*, Experiments, with Observations, on Oleum Terebinthinæ, and its Effects on the Animal Economy . . 107
On the Employment of Terebinthinous Remedies in Disease . 185
DALTON, Mr. JOHN, of *Manchester*, on the Analysis of Mineral Waters . 686
DAVY, Mr. of *Cork*, on a Lactometer . . . . . . 609
DAVY, Dr. of *London*, account of the Leech of Ceylon . . . . 607

PAGE
DE BOUILLON, Dr. of *Guadaloupe*, case of Superfœtation . . . 31
DECANDOLLE, Mr. Mexican Flora . . . . . . . 435
DE LONDE, Mr. of *Paris*, a Dissertation on Gymnastics . . . 56
DESMELLES, Dr. of *Paris*, on a general Transposition of Viscera . 54
DESMOGES, Mr. on three new Vegetable Productions from Senegambia . 517
DOLLINGEN, Professor, on the use of the Spleen . . . . 16
DOWN, Mr. JAMES, of *Leicester*, Observations on the Relations of Heat to Vitality . . . . . . . . . . . 265
DUNN, Mr. JOHN, of *Scarborough*, case of Amputation of part of the Tarsus and Metatarsus, and Preservation of the Shape and Usefulness of the Foot . . . . . . . . . . 504
DUNN, Dr. of *Woolwich*, case of Fracture of the Cranium, with Protrusion of the Brain . . . . . . . 542
FODERÈ, Professor, of *Strasburg*, a Voyage to the Maritime Alps . . 57
FORBES, Dr. of *Penzance*, Medical Topography of Penzance . . 55
FRANK, Dr. LEWIS, of *Parma*, on the use of Black Pepper in Agues . 78
FRANK, Dr. JAMES, Evidence given before a Committee of the House of Commons respecting the Contagion of the Plague . . 123
GEOFFROY ST. HILAIRE, of *Paris*, on Acephalic Fetuses . . . 52
GIBSON, Dr. of the *United States*, on Bronchocele . . . 49
GIMELLE, Dr. of *Paris*, on the Internal Use of Iodine . . . 438
GIRARD, Dr. of *Lyons*, on the use of Liquor Ammoniæ in Drunkenness . 262
GMELIN, Professor, of *Heidelberg*. See TIEDMAN.
GRANVILLE, Dr. of *London*, Note to Dr. Macleod's Paper on Prussic Acid 361
Case of Gastro-enteritis, with Dissections . . . . 364
An account of the Opening of an Egyptian Mummy . . 377
Anti-epileptic Powder . . . . . . . 605
A new Gum-resin . . . . . . . . ib.
GREGORY, Dr. GEORGE, of *London*, Observations on the Scrofulous Inflammation of the Peritoneum in Children . . . . . 497
Case of Malformation of the Heart . . . . . . . 499
Case of Chorea treated by Arsenic . . . . . . ib.
HALFORD, Sir HENRY, Report of the National Vaccine Establishment . 255
HANNAY, Dr. of *Liverpool*, Report of the Liverpool Eye-Infirmary . 598
HAMILTON, Dr. of *Edinburgh*, circulates an account of the Medico-Chirurgical Society of Edinburgh . . . . . . 434
HARRISON, Mr. JOHN, of *London*, account of a case of Obstruction of the Intestinal Canal . . . . . . . . . 177
HARRISON, Dr. EDWARD, of *London*, additional Cases and Remarks illustrative of Spinal Diseases . . . . . . 89
HARRISON, Dr. RICHARD, Evidence given before a Committee of the House of Commons respecting the Contagion of the Plague . 207
HEINRISCH, of *Ratisbon*, on Phosphorescence . . . . . 350
HEUSINGER, Prof. of *Gottingen*, on the Use, and Diseases, of the Spleen 17
HILL, Mr. GEORGE NESSE, of *Chester*, case of Human Rumination . 119
HOWELL, Mr. I. of *Wimbledon*, case of Ossification of the Ventricular Aorta . . . . . . . . . . . 375
HUMBOLDT, Baron, Dissertation on Isothermal Lines . 287, 380, 468, 554
HUME, Mr. of *Long Acre*, case of Poisoning by Arsenic, successfully treated 434, 466, 545
HUSSON, Mr. of *Paris*, Rapport du Comité central de Vaccine en France, for 1818-1819 . . . . . . . . . 508
HUTCHISON, Mr. A. C. of *London*, Remarks on Necrosis, with a Case . 356
Cases of Bronchocele treated by Seton . . . . . 500
HUTCHINSON, Mr. BENJAMIN, of *Southwell*, Observations on the use of Carbonate of Iron in Tic Douloureux . . . . . 284
HUTCHINSON, Dr. W. on Epilepsy . . . . . . . 40
JAMES, Mr. J. H. on the general Principles, and on the particular Treatment, of the different Species of Inflammation . . 225, 317
JEFFREYS, Mr. HENRY, of *London*, Practical Observations on the use of Cubebs, or Java Pepper, in the cure of Gonorrhœa . . 155
JOHNSON, Dr. JAMES, of *Spring Garden*, on the Influence of Tropical Climates on European Constitutions . . . . . . . 507

PAGE

Letter to the *Courier*, on the Epidemia in Spain . . . . 518
A Reply to ditto . . . . . . . . 523
JUKES, Mr. of *London*, Osseous Tumor of the Womb . . . . 56
On Spina Bifida, case announced . . . . . 611
KERNER, Professor, on Poisoning by common Sausage-meat . . 66
KINGLAKE, Dr. of *Taunton*, on the Remedial Power of Turpentine . 272
KNIGHT, THO. ANDREW, esq. of *London*, new Mode of preserving an equable Temperature in the Air of Rooms of Consumptive Patients . 115
LALLEMAND, Dr. of *Montpellier*, on Diseases of the Brain . . 33
LANG, Mr. of *Kensington*, Remarks on Hydrophobia . . . . 257
LASSAIGNE, Mr. of *Alfort*, Appearances observed in a Horse poisoned by Arsenic . . . . . . . . . . 259
On Cathartine, or the active Principle of Senna . . . . 437
LITTLE, Dr. of the *United States*, cases illustrative of the use of Mercurial Ointment in Erysipelas, Swelled Legs . . . . . 280
LIVINGSTONE, Mr. of *China*, account of a Chinese Lusus Naturæ . . 259
LLOYD, Mr. EUSEBIUS ARTHUR, of *London*, a Treatise on the Nature and Treatment of Scrofula . . . . . . . 399
MACAIRE, Mr. of *Geneva*, on the Phosphorescence of the Lampyris Noctiluca 607
MACLEOD, Dr. of *London*, on a new Property of the Hydrocyanic Acid taken internally . . . . . . . . 350
MAIZIERES, Mr. DUDUIT DE, Apple-bread . . . . . 359
MANGIN, Mr. on Vertiginous Indigestion in the Horse . . . 172
MAGENDIE, Dr. of *Paris*, on the Structure of Human Lungs at different Ages 1
On the Lymphatic Vessels of Birds . . . . . . 2
Reptiles and Fishes . . . . 3
On the Means of Absorption . . . . . . 17
On the Non-irritability of Arteries . . . . . . 20
On the Injection of foreign Substances into the Circulation . 21
On a new Mode of treating Hydrophobia . . . . . . 70
Contents of the fourth Number of his Physiological Journal . 611
MAPLESON, Mr. of *London*, a Treatise on the Art of Cupping . . 580
MARLEY, Mr. MILES, of *Vigo-lane*, case of Angina Pectoris . . 453
MARTINET and PARENT, Messrs. of *Paris*, on Diseases of the Arachnoid Membrane . . . . . . . . . . 34
MASSALIEN, Dr. Abstract of Fifty Operations for Hernia . . 610
MAUNOIR, Dr. of *Geneva*, a new Mode of treating Sarcocele . . 71
MAYO, Mr. of *Winchester*, on the Winchester Eye-Infirmary . 434, 399
MECKEL, Professor, of *Halle*, on the Organization of the Heart and Intestines 1
MERCIER and PARANT, of *Quebec*, case of Traumatic Tetanus . . 538
MERREM, Dr. of *Coln*, Experiments on the Tagliacozian Art . . 25
MURRAY, Mr. J. of *London*, Effects of Metallic Salts on Blue Vegetable Colours . . . . . . . . . . . 609
NANCREDE, Dr. of *America*, on Prussic Acid in Pulmonic Diseases . 362
NICHOLL, Dr. WHITLOCK, of *Ludlow*, on disordered States of the Brain in Children . . . . . . . . . . 37
OLMSTED, Professor, of *North Carolina*, case of partial Palsy removed by Lightning . . . . . . . . . . 608
ORFILA, Dr. of *Paris*, on Poisoning by Oxymuriate of Mercury . 68
Leçons faisant partie du cours de Médecine Legale . 158, 244, 337
ORGEL, Mr. on the Presence of Benzoic Acid in the Melilotus Officinalis 516
PARISET, Dr. of *Paris*, Letter on the Epidemy of Spain . . . . 601
PARRY, Captain S. Extract of his Journal of a Voyage of Discovery . 261
Audibility of Sounds . . . . . . . . . 262
PARRY, Dr. CHARLES H. Reply to Dr. Wilson Philip . . . . 100
PELLETIER, Mr. of *Paris*, on Piperine, or the active Principle of Pepper 437
PERCY, Baron, on the Phosphorescence of Wounds . . . . 262
PHILLIPS, Mr. of *London*, on the Effects of Copper on Vegetation . 609
PHYSICIAN, A, of *Edinburgh*, Observations relative to the Origin of the Venereal Disease . . . . . . . . . 258
PHILIP, Dr. A. P. W. of *London*, Treatise on Indigestion, and its Consequences, called Nervous and Bilious Complaints . . 128, 211
PIPER, Mr. S. of *Birmingham*, case of partial Paralysis cured by Urtication 27

PAGE

POWER, Dr. JOHN, Essays on the Female Economy . . . 307
PREVISALI, Dr. of *Pavia*, on the use of Chlorine in Hydrophobia . 597
PRACTITIONER, AN OLD, of *Somersetshire*, on the Frauds practised by Persons to gain Admission to the Medical Profession . . 171
PRICE, Mr. DAVID, case of Sudden Death, in which a Hydatid was found in the Substance of the Heart . . . . . . . 502
PRING, Mr. of *Bath*, on the Formation of artificial Anus . . 72
PURTON, Mr. THOMAS, of *Alcester*, on Ruptures of the Uterus . 451
Extraordinary Case of Distension of the Œsophagus . . 540
REEDER, Dr. HENRY, of *London*, a Practical Treatise on Diseases of the Heart . . . . . . . . . . 569
REID, Dr. of *London*, Essays on Hypochondriasis . . . . . 560
RICHMOND, Mr. of *Grimsby*, case illustrative of the beneficial Effects of the Carbonate of Iron . . . . . . . . . 270
RIGBY, Dr. of *Norwich*, an account of his Death . . . . 612
RUST, Dr. on the Egyptian Ophthalmia . . . . . . 41
On Hospital Gangrene . . . . . . . . 75
SARLANDIÈRE, Dr. of *Paris*, case of Delirium produced by Belladonna and Stramonium, presenting the character of Somnambulism . 120
SAVI, Dr. PAOLO, of *Pisa*, on a new Species of Salamander . . 597
SCOTT, Mr. P. N. of *Norwich*, case of Separation of a Portion of the Uterus during severe Labour . . . . . . . . 506
SCHREGER, Professor, of *Erlangen*, on a new species of Mucous Follicle under the Skin . . . . . . . . . 8
SCUDAMORE, Dr. of *London*, Reply to Mr. Bampfield's Observations on the use of Colchicum . . . . . . . 551
SERRES, Dr. of *Paris*, on the Comparative Anatomy of the Brain . 81
SHAW, Mr. of *London*, a Manual for the Student of Anatomy . . 416
SKENE, Dr., and Dr. EWING, of *Aberdeen*, Galvanic Experiments on a Criminal . . . . . . . . . . . 612
SMERDON, Mr. of *Clifton*, case of Peripneumonia and Arteritis . . 529
SMITH, Mr. of *Bristol*, Reply to the Remarks of Mr. Tripe on the Statements in his Statistic Inquiry into the frequency of Stone in the Bladder . . . . . . . . . . . 117
SUNDERLAND, Dr. of *Barmen*, on the Nutrition of the Fetus . . 31
SWAN, Mr. JOSEPH, of *Lincoln*, on the Physiology of the Ear . . 503
TIEDMAN, Professor, of *Heidelberg*, on the use of the Spleen . . 12
On the Influence of the Thoracic Duct on the Circulation . 11
On a Communication between the Absorbents of the Intestines and the Vena Portarum . . . . . . . . 13
TORDEUX, Mr. of *Cambrai*, Nitrate of Potash in the Cochlearia Officinalis 517
TRIPE, Mr. of *Plymouth Dock*, a Reply to Mr. Smith's Observations on the frequency of Calculous Disorders in Great Britain . . 371
TURNBULL, Mr. British Consul at *Marseilles*, official Documents respecting the Importation of Yellow-Fever in the Lazaretto of Marseilles
URE, Dr. ANDREW, of *Glasgow*, a Dictionary of Chemistry . . 241
VACCA, Dr. ANDREA BERLINGHIERI, on the Operation of Lithotomy by the Rectum . . . . . . . . . . 430
VAN REES, Mr. on the Propagation of Sound in Elastic Fluids . . 608
VALLÈE, Mr. of *Paris*, on the Theory of Vision . . . . 26
VALLOT, Mr. on a Plant soluble in Water . . . . . 609
VAUQUELIN, Mr. Decomposition of Blood . . . . . 251
WARD, Mr. H. of *Maidenhead*, on Tar Fumigation . . . 454
WERNECK, Dr. of *Vienna*, on Hospital Gangrene . . . . 41
WILSON, Dr. of *America*, on the Tincture of the Poppy as a Substitute for Laudanum . . . . . . . . . . 544
WILSON, Mr. JAMES, of *London*, Lectures on the Structure, Physiology, and Diseases of the Male Urinary Organs . . . . . 476
An account of his Death . . . . . . . . 607
WURZA, Mr. Action of Cork on Chalybeate Water . . . . 259
YEATMAN, Mr. J. CHARLETON, of *Frome*, a Reply to an Old Practitioner on the Frauds practised by Persons to gain Admission into the Medical Profession . . . . . . . . 535

THE LONDON
### Medical and Physical Journal.

1 OF VOL. XLVI.] JULY, 1821. [NO. 269.

### NOTICE.

*Another of the series of* PRÖEMIA *to the several volumes of this Journal, (which commenced with that to the forty-third volume,) comprising a History of the Progress of Medicine and its auxiliary Sciences for the half-year immediately previous to the period of their production, respectively, will be published on the last day of July. One of the especial intentions of those* Pröemia, *is to present a comprehensive view of the state and progress of Medicine throughout Europe generally, and in the United States of America; an object that cannot be effected in the regular monthly Numbers of the Journal, because of the small extent of space which can be there appropriated to this purpose.*

### MONTHLY CATALOGUE OF MEDICAL BOOKS.

A Series of Questions and Answers, for the Use of Gentlemen preparing for their Examination at Apothecaries' Hall; with copious and useful Tables annexed. By C. M. Syder. 12mo. 4s.

Observations on some of the general Principles, and on the particular Nature and Treatment of the different Species of Inflammation. By J. M. James. 8vo. 10s. 6d.

Practical Observations on the Use of the Cubebs, or Java Pepper, in the Cure of Gonorrhœa. By Henry Jeffreys. 8vo. 3s.

Barker and Cheyne on the Epidemic Fever of Ireland. 2 vols. 8vo. 1l. 6s.

Griffith on Vertebrated Animals. 4to. Part I. 35 Plates. 1l. 5s.

A Treatise on Scrophula, (to which the Jacksonian Prize for the year 1818 was adjudged by the Court of Examiners of the Royal College of Surgeons.) By Eusebius Arthur Lloyd, Member of the Royal College of Surgeons in London, &c. 8vo. 9s.

Practical Observations on Cold and Warm Bathing. By James Millar, M.D. 12mo. 4s. 6d.

A Syndesmological Chart, or a Table of the Ligaments of the Human Skeleton. By J. Dickinson, M.D. 1s.

A Series of Lectures on Modern Surgery. By C. M. Syder. 8vo. 12s.

[HIGHLEY AND SON, FLEET-STREET.]

